# Supplementary material for: Plasmodium falciparum immunodominant IgG epitopes in subclinical malaria
Source: Sci Rep. 2020 Jun 10;10:9398. doi: 10.1038/s41598-020-66384-0 (PMC7287129; doi:10.1038/s41598-020-66384-0)
Supplement: Supplementary file 1 — Supplementary Information. [file 41598_2020_66384_MOESM1_ESM.docx]

**SUPPLEMENTARY INFORMATION FOR:**

**Plasmodium falciparum immunodominant IgG epitopes**

**in subclinical malaria**

Isabel G. Azcárate, Patricia Marín-García, Paloma Abad, Susana Pérez-Benavente, Estela Paz Artal, Pedro A. Reche, Julius N. Fobil, José M. Rubio, Amalia Diez, Antonio Puyet & José M. Bautista

Correspondence:

José M. Bautista ([jmbau@ucm.es](mailto:jmbau@ucm.es)) & Antonio Puyet ([apuyet@ucm.es](mailto:apuyet@ucm.es))

**This PDF file includes:**

Supplementary text

Figures S1 to S2

Tables S1 to S4

SI References

SUPPLEMENTARY TEXT

Material and Methods for data shown in Supplementary Table S2 and Supplementary Table S2.

**Detection of Plasmodium spp. (Tables S1, S2 and S3)**

Presence or absence of human malaria parasites in blood samples from imported malaria cases was detected in a real-time PCR assay targeting the species-specific sequences of the 18S ribosomal DNA (rDNA) of *P. falciparum*, *P. ovale*, and *P. malariae* (1). To know the efficiency of the PCR reaction, the amplification of a fragment of the gene coding for human GADPH using commercial primers (GADPH TaqMan® gene expression assays 20X, Thermofisher Scientific) was performed simultaneously for each of the samples. Samples of imported malaria patients were selected if positive amplifications to only 18S rRNA from *P. falciparum* were displayed. Samples amplifying other Plasmodium species 18S rRNA were discarded.

*P. falciparum* parasite load (in %) in samples from Breman-Asikuma was determined by real time PCR using quantified lab-cultures parasites. Each reaction plate included five serial dilutions (0.5, 0.1, 0.05, 0.025, 0.005 % parasitemia) of *P. falciparum* genomic DNA extracted from lab-cultured parasites (2) as positive controls, and including a negative control with molecular-grade water instead of DNA, all in duplicate. For each plate, threshold lines were set manually and mean Ct was calculated for each amplified duplicate. Ct values were plotted against the logarithmic parasitemia values of *P. falciparum* control gDNA to calculate correlation coefficient values and parasitemia percentages. The sensitivity of this PCR assay can detect submicroscopic infections (≈0.005 % parasitemia in P. falciparum).

**HLA genotyping (Table S2)**

DNA extraction from blood spots of people from endemic malaria areas was carried out following the protocol InstaGene Whole Blood kit (Bio-Rad Laboratories). Briefly, blood spots on Whatman protein saver cards were soaked overnight in a tube containing 100 µL of phosphate-buffered saline (PBS) at 4°C. The tube was centrifuged at 11,000 x g for two minutes. After discarding the supernatant, 100 µL of PBS was added to wash the sediment similarly. The sediment was vortexed for 15 seconds and incubated with 60 µL of InstaGene Matrix CHELEX (Bio‐Rad) for 4 minutes at 100ºC. After vortexing again, the tube was centrifuged at 11,000 x g for one minute and the supernatant was aspirated to be purified once again as described above with InstaGene Matrix. The supernatant was then used for the PCR. Aliquots of these DNA samples (20 μl) were taken and HLA-typed by the LABType SSO Class II DRB1 (One Lambda) kit.

For peptide selection, we sought to identify 15-mer peptides in the selected five antigens that could potentially be detected by B-cells and presented by HLA II molecules incorporating HLA-DRB1 alleles. Peptide presentation by HLA-II molecules was assessed after peptide-binding predictions to a panel of HLA-DRB1 molecules expressed by the malaria patient cohort using NetMHCIIpan (3). Only high binders were considered. Subsequently, we selected the minimum set of peptides that for exhibiting promiscuous HLA II peptide-binding covered the all the HLA-DRB1 alleles in the cohort.

Fig. S1.


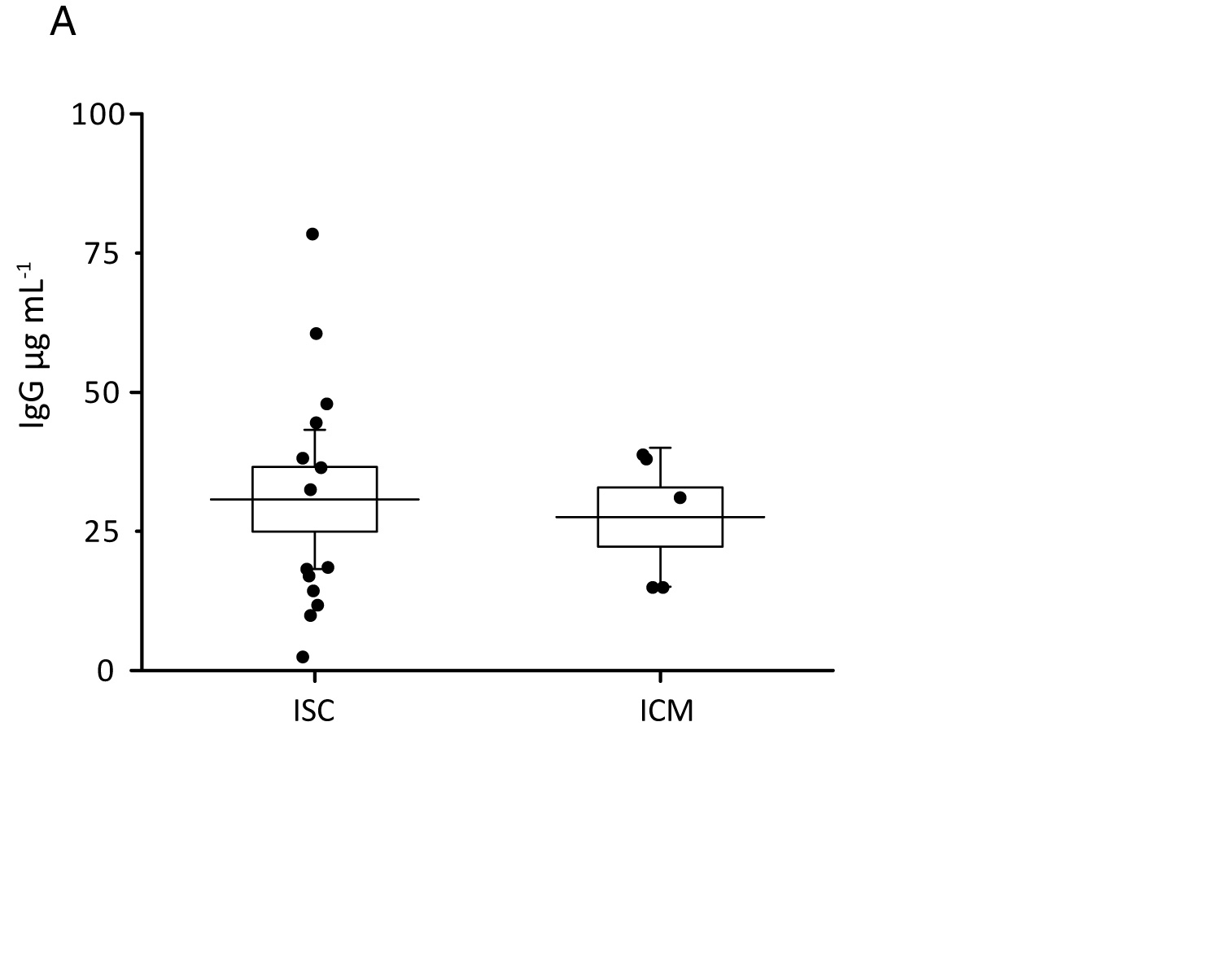


**Figure S1. Concentration of anti-P. falciparum IgG antibody and IgG avidity in sera of imported malaria patients.** A) IgG concentrations obtained by indirect ELISA using immobilized *P. falciparum* total protein as target. Each dot is the IgG concentration of a patient. Horizontal line shows the mean value covered with a box depicting the mean standard error. Whiskers shows the 95% confidence interval.

**Figure S2**

**Figure S2. Two-dimensional electrophoresis profile of *P. falciparum* crude extracts stained with SyBR Pro Ruby and the common IgG reactive proteins detected with sera from imported malaria patients.** A) Total *P. falciparum* proteins separated by 2D gel electrophoresis using a linear pI 3–11 1st dimension. Gels were SYPRO Ruby stained. B) Representative replica gels western blotted with patient sera from ISC and ICM groups. Spots that were recognized by at least 30% of patient sera on parallel western blots were selected for identification (circled in red at panel A). Spots whose identity was determined are numbered in panel A and depict the proteins described in Table 1. See Material and Methods section in main text.

**Table S1.** Demographics and IgG values of imported malaria sera samples with *P. falciparum* detected parasitemia used in this study.

| **Group^a^** | **Sample Id.** | **Sex** | **Age (years)** | ***Place of origin*** | ***Pf-specific IgG μg/mL*** |
| --- | --- | --- | --- | --- | --- |
| ISC | 1517 | M | 59 | Equatorial Guinea | 61 |
|  | 5517 | M | 28 | Unknown | 12 |
|  | 38708 | F | 36 | Equatorial Guinea | 17 |
|  | 42350 | F | 45 | Equatorial Guinea | 10 |
|  | 44270 | M | 53 | Equatorial Guinea | 18 |
|  | 46130 | F | 64 | Equatorial Guinea | 79 |
|  | 48751 | F | 57 | Equatorial Guinea | 38 |
|  | 48790 | F | 45 | Equatorial Guinea | 32 |
|  | 5637 | M | 31 | Cameroon | 2 |
|  | 39732 | F | 40 | Equatorial Guinea | 19 |
|  | 42098 | M | 73 | Equatorial Guinea | 14 |
|  | 49039 | M | 45 | Equatorial Guinea | 36 |
|  | 52746 | M | 38 | Equatorial Guinea | 45 |
|  | 52899 | F | 69 | Equatorial Guinea | 48 |
| ICM | 6031 | M | 34 | Conakry Guinea | 15 |
|  | 35421 | F | 29 | Equatorial Guinea | 39 |
|  | 4827 | M | 23 | Cameroon | 31 |
|  | 5256 | F | 50 | Undeclared | 15 |
|  | 6032 | M | 18 | Senegal | 38 |

**^a^** ISC: Imported subclinical malaria group, parasitemia <0.01%; ICM: Imported clinical malaria group.

**Table S2.** Demographics and IgG values of sera samples collected at Breman-Asikuma from adults used in this study.

| **Group^a^** | **Sample Id.** | | **Sex** | | **Age (years)** | | ***Pf*-specific IgG μg/mL^b^** | | **Anti-*Pf* IgG High/Low^c^** | |
| --- | --- | --- | --- | --- | --- | --- | --- | --- | --- | --- |
| ASC | 284 | F | | 28 | | 4 | | L | |  |
|  | 64* | F | | 25 | | 8 | | L | |  |
|  | 88* | F | | 30 | | 10 | | L | |  |
|  | 135 | M | | 26 | | 10 | | L | |  |
|  | 253 | M | | 26 | | 16 | | L | |  |
|  | 25 | F | | 33 | | 22 | | L | |  |
|  | 296* | M | | 27 | | 22 | | L | |  |
|  | 86 | F | | 31 | | 25 | | L | |  |
|  | 61* | F | | 17 | | 26 | | L | |  |
|  | 40 | M | | 30 | | 45 | | L | |  |
|  | 225 | F | | 26 | | 59 | | L | |  |
|  | 259 | F | | 29 | | 63 | | H | |  |
|  | 288* | F | | 28 | | 63 | | H | |  |
|  | 145 | M | | 35 | | 73 | | H | |  |
|  | 22* | M | | 21 | | 77 | | H | |  |
|  | 66 | M | | 30 | | 84 | | H | |  |
|  | 60 | M | | 28 | | 85 | | H | |  |
|  | 62 | F | | 30 | | 102 | | H | |  |
|  | 300* | M | | 29 | | 160 | | H | |  |
|  | 307 | F | | 33 | | 225 | | H | |  |
|  | 193* | F | | 25 | | 692 | | H | |  |
| ANP | 281* | F | | 26 | | 14 | | L | |  |
|  | 310 | M | | 40 | | 14 | | L | |  |
|  | 230 | M | | 23 | | 17 | | L | |  |
|  | 290* | M | | 20 | | 20 | | L | |  |
|  | 100 | M | | 28 | | 23 | | L | |  |
|  | 314 | M | | 28 | | 27 | | L | |  |
|  | 125* | F | | 19 | | 39 | | L | |  |
|  | 141* | M | | 22 | | 52 | | L | |  |
|  | 9* | F | | 25 | | 65 | | H | |  |
|  | 247 | M | | 21 | | 67 | | H | |  |
|  | 132 | F | | 26 | | 72 | | H | |  |
|  | 302 | F | | 22 | | 84 | | H | |  |
|  | 144 | F | | 26 | | 89 | | H | |  |
|  | 209* | F | | 20 | | 105 | | H | |  |
|  | 270* | M | | 19 | | 117 | | H | |  |
|  | 271 | M | | 32 | | 130 | | H | |  |
|  | 4* | F | | 26 | | 145 | | H | |  |

**^a^** ASC: Adult subclinical malaria group, parasitemia <0.01%; ANP: undetected parasitemia group

**^b^** Average ratio Pf IgG/Total IgG for L samples: 0.012±0.008, H samples: 0.050±0.035 (p=0.0003).

**^c^** Cut off for Hig/Low IgG content 60 μg mL^-1^

* The samples labeled with asterisk were selected for the validation of the 20-mer immunoreactive epitopes (Figure 3).

**Table S3.** Demographics and parasitemia of sera samples collected at Breman-Asikuma from children under 5 y.o. used in this study.

| **Group^a^** | **Sample Id.** | **Age (months)** | **Parasitemia (%)** |
| --- | --- | --- | --- |
| CCM | 91 | 60 | 6.50 |
|  | 210 | 18 | 3.45 |
|  | 71 | 36 | 7.65 |
|  | 297 | 18 | 0.02 |
|  | 77 | 12 | 0.02 |
|  | 95 | 48 | 0.33 |
|  | 124 | 36 | 1.48 |
| CSC | 260 | 13 | 0.005 |
|  | 298 | 18 | 0.005 |
|  | 10 | 60 | <0.005 |
|  | 94 | 60 | <0.005 |
|  | 303 | 48 | <0.005 |
|  | 215 | 24 | <0.005 |
|  | 167 | 36 | <0.005 |
| CNP | 201 | 48 | U.D. |
|  | 208 | 1.5 | U.D. |
|  | 150 | 7 | U.D. |
|  | 105 | 8 | U.D. |
|  | 265 | 36 | U.D. |
|  | 53 | 48 | U.D. |

^a^ CCM: Children Clinical Malaria group, parasitemia >0.01%; CSC: Children Subclinical Malaria group, parasitemia <0.01%; CNP: Children with undetected parasitemia. U.D.: Undetectable. CCM group at admission showed fever, malaise and chills as only symptoms.

Average age (months) at each group: CCM= 32.6±17.6; CSC=37.0±19.5; CNP=24.8±21.6. Not significant differences among groups.

**Table S4.** HLA genotypes of sera samples collected at Breman-Asikuma from adults used in this study.

| **Group^a^** | **Sample Id.** | **Assigned Allele Pair** | **Assigned Sero** |
| --- | --- | --- | --- |
| ASC | 284 | DRB1*08:04 DRB1*09:01 | DR8 DR9 |
|  | 64 | DRB1*07:01 DRB1*09:02 | DR7 DR- |
|  | 88 | U.D. | U.D. |
|  | 135 | DRB1*10:01 DRB1*13:02 | DR10 DR13 |
|  | 253 | DRB1*08:04 DRB1*14:01 | DR8 DR14 |
|  | 25 | U.D. | U.D. |
|  | 296 | DRB1*07:01 DRB1*08:04 | DR7 DR8 |
|  | 86 | DRB1*13:02 DRB1*13:24 | DR13 DR- |
|  | 61 | DRB1*03:02 DRB1*04:05 | DR18 DR4 |
|  | 40 | DRB1*01:02 DRB1*14:123 | DR1 DR- |
|  | 225 | U.D. | U.D. |
|  | 259 | DRB1*03:02 DRB1*13:02 | DR18 DR13 |
|  | 288 | DRB1*11:01 DRB1*13:02 | DR11 DR13 |
|  | 145 | DRB1*08:01 DRB1*11:04 | DR8 DR11 |
|  | 22 | DRB1*07:01 DRB1*14:01 | DR7 DR14 |
|  | 66 | U.D. | U.D. |
|  | 60 | U.D. | U.D. |
|  | 62 | DRB1*10:01 DRB1*13:02 | DR10 DR13 |
|  | 300 | U.D. | U.D. |
|  | 307 | U.D. | U.D. |
|  | 193 | U.D. | U.D. |
| ANP | 281 | DRB1*01:02 DRB1*03:02 | DR1 DR18 |
|  | 310 | U.D. | U.D. |
|  | 230 | U.D. | U.D. |
|  | 290 | DRB1*03:02 DRB1*15:03 | DR18 DR15 |
|  | 100 | DRB1*01:02 DRB1*08:04 | DR1 DR8 |
|  | 314 | U.D. | U.D. |
|  | 125 | DRB1*10:01 DRB1*13:01 | DR10 DR13 |
|  | 141 | DRB1*07:01 DRB1*13:03 | DR7 DR13 |
|  | 9 | DRB1*01:02 DRB1*13:02 | DR1 DR13 |
|  | 247 | DRB1*01:02 DRB1*08:04 | DR1 DR8 |
|  | 132 | DRB1*09:01 DRB1*16:21N | DR9 DR"Blank" |
|  | 302 | DRB1*11:01 DRB1*15:03 | DR11 DR15 |
|  | 144 | DRB1*07:01 DRB1*08:04 | DR7 DR8 |
|  | 209 | U.D. | U.D. |
|  | 270 | DRB1*15:03 DRB1*16:02 | DR15 DR16 |
|  | 271 | DRB1*03:02 DRB1*13:02 | DR18 DR13 |
|  | 4 | DRB1*01:02 DRB1*04:01 | DR1 DR4 |

**^a^** ASC: Adult subclinical malaria group, parasitemia <0.01%; ANP: undetected parasitemia group

U.D.: Undetermined

**REFERENCES**

1. A. M. Rantala *et al.*, Comparison of real-time PCR and microscopy for malaria parasite detection in Malawian pregnant women. *Malar J* **9**, 269 (2010).

2. A. Radfar *et al.*, Synchronous culture of *Plasmodium falciparum* at high parasitemia levels. *Nat Protoc* **4**, 1899-1915 (2009).

3. M. Nielsen *et al.*, Quantitative predictions of peptide binding to any HLA-DR molecule of known sequence: NetMHCIIpan. *PLoS Comput Biol* **4**, e1000107 (2008).
